# The burden of pain in rheumatoid arthritis: Impact of disease activity and psychological factors

**DOI:** 10.1002/ejp.1651

**Published:** 2020-09-11

**Authors:** Pascale Vergne‐Salle, Sophie Pouplin, Anne Priscille Trouvin, Anne Bera‐Louville, Martin Soubrier, Christophe Richez, Rose Marie Javier, Serge Perrot, Philippe Bertin

**Affiliations:** ^1^ Department of Rheumatology and Pain Center Centre Hospitalier Universitaire Limoges France; ^2^ Department of Rheumatology and Pain Center Centre Hospitalier et Universitaire Rouen France; ^3^ Pain Center Hôpitaux Universitaires Paris Centre Cochin Paris France; ^4^ Department of Rheumatology Centre Hospitalier Universitaire Lille France; ^5^ Department of Rheumatology Centre Hospitalier Universitaire Clermont‐Ferrand France; ^6^ Department of Rheumatology Centre Hospitalier Universitaire Bordeaux France; ^7^ Department of Rheumatology Centre Hospitalier Universitaire Strasbourg France

## Abstract

**Background:**

Pain remains a prevalent symptom for rheumatoid arthritis (RA) patients despite a wide therapeutic choice. The objective of this study was to provide a multidimensional evaluation of pain.

**Methods:**

A total of 295 RA patients from 7 French rheumatology centres were enrolled in a cross‐sectional study. Patients completed a chronic pain assessment questionnaire approved by the French National Authority for Health, the health assessment questionnaire (HAQ) as well as depression and anxiety scales (HAD, Beck Depression Inventory, STAI). Disease activity (DAS28) and ESR were recorded. A multivariate descriptive analysis was undertaken using principal component analysis (PCA).

**Results:**

38.4% of patients had a pain score > 40 mm/100, although 83% were on biological treatment and 38.7% were in remission based on the RA activity score. The PCA analysis found four axes representing 70% of total variance. The axes, per cent of variance and variables represented were as follows: (a) axis 1, 41% variance, anxiety and depression scores, sensory and affective qualifier score, HAQ and pain impact on daily life; (b) axis 2, 13% variance, disease activity score (DAS28) and pain relief with current treatment; (c) axis 3, 9% of variance, RA duration and radiographic score and (d) axis 4, 6% of variance, DAS28 and ESR. Moderate to severe pain was significantly associated with axes 1 and 2.

**Conclusions:**

Despite a high proportion of patients on biological treatments, 38.4% of patients continue to experience moderate to severe pain. Pain is associated with the RA activity score, but also with the depression and anxiety scores.

**Significance:**

Substantial proportion of rheumatoid arthritis (RA) patients still experiences relevant pain, although more than 80% on biological treatment. Pain is primarily associated with anxiety and depression scores and with disease activity score. These findings highlight the need to assess patients’ mental well‐being alongside. Clinical measures of disease activity to better manage pain and guide treatment decisions.

## 1 INTRODUCTION

Pain and disability are prevalent complaints in rheumatoid arthritis (RA). Inflammation and joint destruction are traditionally considered to cause nociceptive pain, but the intensity of the pain does not always correlate with the disease activity score (DAS) and structural damages. In recent years, there have been many improvements in RA treatment with the emergence of biological Disease‐Modifying Anti‐Rheumatic Drugs (bDMARDs) frequently associated with synthetic DMARDS (sDMARDs) (e.g. methotrexate). Fewer patients have severe inflammatory and destructive disease. Nevertheless, across multiple areas of health measures, pain relief remains a priority for patients (Heiberg & Kvien, 2002; Sanderson, Morris, Calnan, Richards, & Hewlett, 2010). Pain remains a concern for as many as around 40% of patients taking bDMARDs and 51.6% taking sDMARDs (Sanderson et al., 2010).

In the RAID study (Gossec et al., 2009) and Hewelett et al.'s (2005) survey, the pain experienced by patients was in the top two outcomes along with aspects of physical function. After 6 months of treat to target strategy in early RA, 40.2% of patients did not achieve an absolute patient‐perceived satisfactory improvement in pain (improvement ≥ 30 mm in VAS pain) (Ten Klooster et al., 2015). Persistent Pain in RA is a complex and multifactorial phenomenon, due to peripheral inflammation and sensitization, but also to central pain mechanisms with central sensitization (Lee et al., 2018; Vladimirova et al., 2015). Using the pain DETECT questionnaire, about 20% of RA patient had pain with neuropathic characteristics (Koop, ten Klooster, Vonkeman, Steunebrink, & van de Laar, 2015; Rifbjerg‐Madsen et al., 2017). The emotional dimensions and consequences the disease may have on quality of life can have a significant impact on pain intensity experienced by patients. Lee et al. have found three subgroups of patients based on pain, inflammation, fatigue and psychosocial factors. Among them, one subgroup was characterized by minimal inflammation but high pain, fatigue and psychosocial distress (Lee et al., 2014). Comorbid fibromyalgia, with a pooled prevalence of 20% in RA, may explain the disconnect between persistent pain and improvement in inflammation, but it is not probably the single mechanism (Lee, 2013; Zhao, Duffield, & Goodson, 2019). Wolfe and colleagues have defined the term of fibromyalgianess in RA patients with chronic pain and other somatic symptoms, who did not satisfy the fibromyalgia criteria (Wolfe et al., 2014).

Immune cells and their mediators (cytokines) have an emerging role in pain regulation (Ji, Chamessian, & Zhang, 2016; Raoof, Willemen, & Eijkelkamp, 2018). RA treatments, especially bDMARDs with their cytokine‐blockade effects, may have pain‐reducing properties apart from their anti‐inflammatory and structural effects (Hess et al., 2011).

The objectives of this study were to: (a) Carry out an assessment of pain and of its various components in RA patients managed in a hospital setting with current treatments and without fibromyalgia, (b) Assess the links between different RA characteristics, emotional state, functional state and pain and (c) Compare the effects of different DMARDS on pain.

## 2 METHODS

This is a cross‐sectional, multicenter, descriptive observational study conducted in seven French rheumatology centres (NCT01706029).

### 2.1 Study population

Patients willing to participate in the study were recruited in the course of in‐ or outpatient care between October 2012 and December 2014. The study was conducted following approval of a French regional Ethics Committee and all patients gave their written informed consent prior to their inclusion in the study.

To be eligible for the study, patients had to have established RA according to the 1987 ARA criteria (Arnett et al., 1988). They had to be treated with sDMARDs (methotrexate, leflunomide, sulfasalazine) and/or bDMARDs (anti‐TNF‐α, rituximab, tocilizumab or abatacept) for a minimum of 3 months. Analgesics, non‐steroidal anti‐inflammatory drugs (NSAIDs) or corticosteroids had been prescribed at a stable dose for at least 2 weeks.

Non‐inclusion criteria included the presence of a painful pathology other than RA that could interfere in the assessment of pain (including fibromyalgia), progressive cancer or lymphoproliferative disease, severe psychiatric conditions and dementia, acute infectious diseases or being less than 18 years of age.

Patients with fibromyalgia (with the 1999 ACR criteria) were excluded to not interfere with pain and psychological assessments.

### 2.2 Assessment criteria

The main outcome was the multidimensional assessment of pain carried out using a chronic pain assessment questionnaire for adults, as approved by the French National Authority for Health (HAS) (https://has‐sante.fr/upload/docs/application/pdf/douleur1.pdf), a questionnaire containing elements of the Brief Pain Inventory (Tan, 2004) and the short‐form McGill Pain Questionnaire (Melzack, 2005). This questionnaire was completed only once by the patient, at the time of inclusion in the study. This multidimensional questionnaire included:

- A visual analogue scale (VAS) of 100 mm or a numeric scale (NS) from 0 to 10 (if VAS was not comprehensible) to assess the pain currently experienced, the average pain intensity experienced over the last 8 days (VAS8d) and the most intense pain experienced over the last 8 days.
- Different pain qualifiers (short‐form Mc Gill Pain questionnaire) to measure pain experienced (9 of them sensory and 7 emotional), with a scale for each ranging from 0 (absent), through 1 (weak), 2 (moderate) and 3 (strong) to 4 (extremely strong). The scores have been expressed as percentages of the total score for each of the two categories.
- The Hospital Anxiety and Depression Scale (HAD) (scores range from 0 to 21, ≤ 7: no disturbance, between 8 and 10: doubtful cases, ≥ 11: anxiety or depression).
- A scale for assessing the interference of pain in daily life including 6 areas (mood, walking ability, normal work, relationships with others, sleep and enjoyment of life), each with a scale of 0 to 10.
- Evaluation of pain relief with current treatment (VAS or NS: no relief or 0 to maximal relief or 100)

The secondary outcomes were as follows:

- A verbal scale to measure patient satisfaction with pain management.
- The RA disease activity score: DAS28‐ESR administered by trained physician
- The Stanford Health Assessment Questionnaire (HAQ) (scores range from 0 to 3).
- Short‐form of Beck depression Inventory (BDI) (13 items, score range from 0 to 39; 0 to 4 = no depression, 5 to 7: mild, 8 to 15: moderate, ≥16: severe depression) (Beck & Steer, 1984).
- The State‐Trait Anxiety Inventory (STAI) with a STAI‐state score (STAI‐A) and a STAI‐trait score (STAI‐B) (scores range from 0 to 80 for each) (Spielberger, 2010).
- A structural evaluation of articular destructions with SENS score (score ranging from 0 to 86) (van der Heijde, Dankert, Nieman, Rau, & Boers, 1999) calculated on the basis of x‐rays of the hands and forefeet taken within the previous 6 months.
- Current treatments including sDMARDs, bDMARDs, NSAIDs, steroids, conventional painkillers (acetaminophen, opioids) and treatment histories for sDMARDs and bDMARDs.

### 2.3 Statistical analysis

Statistical analysis was carried out by the Center for Epidemiology, Biostatistics and Research Methodology (CEBIMER) of the Limoges University Hospital using SAS^®^ 9 software (SAS Institute 3 Cary, NC) and R v3.3.2 ("PMCMR" v4.1, "FactoMineR" v1.35 packages). The STROBE recommendations were followed (von Elm et al., 2008). The significance threshold for all analysis was set at 0.05.

A descriptive analysis of the data was performed first in the total sample then in subgroups: (a) patients with VAS8d < 40 mm versus ≥40 to separate patients with moderate and severe pain from those with only mild pain (Tubach et al., 2012), (b) type of sDMARDs/ bDMARDs treatment and (c) types of bDMARDs (5 categories: sDMARDs alone, anti‐TNF ± sDMARDs, abatacept ± sDMARDs, rituximab ± sDMARDs and tocilizumab ± sDMARDs). Quantitative variables have been presented as mean ± standard deviation, and qualitative variables expressed in absolute and percentage terms. Comparisons of the qualitative variables were carried out using Chi‐square tests or Fisher exact tests depending on the application conditions. The comparisons of the quantitative variables were made by Student or Mann–Whitney tests depending on the distribution evaluated and by the Kruskal–Wallis tests and post hoc adapted tests with a Bonferroni correction applied to avoid the inflation of alpha risk for comparisons between more than two groups. Normality was evaluated graphically and by using Shapiro–Wilks tests.

As part of the multidimensional description of pain, principal component analysis (PCA) was computed on all explanatory highly correlated quantitative variables with VAS8d (<40, ≥40) as an illustrative variable. Two complementary analyses were also computed with (a) "the most intense pain experienced in the previous 8 days" (<40, ≥40) and (b) "current pain" (<40, ≥40) as illustrative variables.

A one‐way analysis of variance was performed for each new axis to compare the coordinates of the individuals on this axis differed depending on the illustrative variable studied (<40, ≥40). *T*‐test was also performed by modality of the illustrative variable studied to determine whether the coordinates of the individuals on the axis varied significantly from the average coordinates.

For the following variables: current pain VAS, Last 8 days average pain VAS and Worse pain VAS, if some data were missing, they were replaced by the associated pain numeric scale (0 to 10) multiplied by 10 to have a score on 100.

## 3 RESULTS

A total of 299 RA patients were eligible; 295 were included and completed the study, 3 patients did not meet the inclusion criteria and 1 patient withdrew consent. Seventy‐two patients were not eligible because they fulfilled the 1999 ACR fibromyalgia criteria.

### 3.1 Characteristics of the study population

Patient demographics and RA characteristics are shown in Table 1. Regarding RA activity, 38.7% of patients were in remission (DAS28 ≤ 2.6), 15.4% in low activity (DAS28 [2.7–3.2]), 37.7% active (DAS28 [3.3–5.1]) and 8.2% in high activity (DAS28 > 5.1). The treatments (analgesics, anti‐inflammatory drugs, steroids and DMARDs) are summarized in Table 1.

**TABLE 1:** Patients characteristics (*N* = 295)

	Mean or %	*SD* or *N*
Age (years) (*N* = 295)	58.4	11.8
Gender (female) (*N* = 295)	80.3%	237
Disease duration (years) (*N* = 294)	13.3	9.6
Rheumatoid factor positivity (*N* = 280)	76.4%	214
ACPA positivity (*N* = 270)	74.1%	200
CRP (mg/l) (*N* = 291)	6.0	9.6
Number of swollen joint (*N* = 295)	2.5	3.1
Number of tender joint (*N* = 295)	3.4	4.7
ESR (mm/h) (*N* = 292)	14.0	14.7
DAS28‐ESR (*N* = 292)	3.1	1.4
SENS score (*N* = 274)	21.5	19.7
HAQ score (*N* = 292)	1.1	0.7
Treatment (*N* = 295)		
Analgesic treatments	66.1%	195
Acetaminophen	64.1%	125
Weak opioids	45.6%	89
Strong opioids	7.2%	14
NSAIDs	24.4%	72
Steroids	42.7%	126
Dose (mg/d)	6.4	4.9
Synthetic DMARDs	69.1%	204
Biologic DMARDs	83.0%	245
AntiTNF	22.0%	54
Abatacept	23.7%	58
Rituximab	16.3%	40
Tocilizumab	38.06%	93

Note

Rheumatoid factor positivity (>ULN = 15 IU/ml).

Abbreviations: ACPA, anti‐citrullinated protein antibody (>ULN = 25 U/ml); CRP, C‐reactive protein (mg/l); DAS28‐ESR, disease activity score for 28 joint counts based on the erythrocyte sedimentation rate; DMARDs, disease‐modifying anti‐rheumatic drugs; HAQ, Health Assessment Questionnaire from 0 to 3; NSAIDs, non‐steroidal anti‐inflammatory drugs; SENS, radiographic score (simple erosion narrowing score).

### 3.2 Pain analysis

**FIGURE 1:**
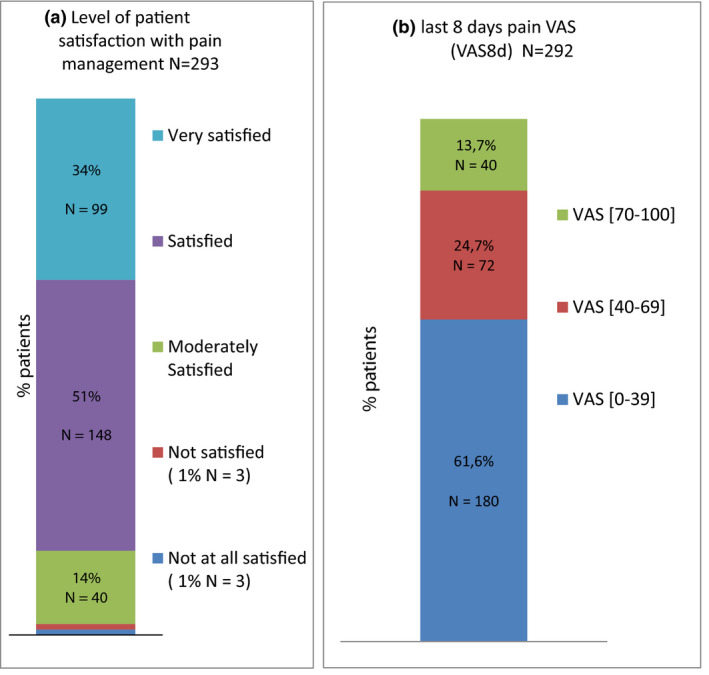
(a) Previous 8 days average pain VAS for the 295 RA patients, classified as no pain or slight pain (VAS between 0 and 39 mm/100), moderate pain (VAS between 40 and 69 mm/100 mm) and severe pain (VAS between 70 and 100 mm/100 mm); (b) level of patient satisfaction with pain management

In terms of pain management, satisfaction was generally good, as shown in Figure 1a.

However, a high percentage of patients (38.4%) had a VAS8d ≥ 40 mm/100, while 13.7% had a VAS8d ≥ 70 mm/100 (Figure 1b). The mean VAS8d was 33.6 ± 26.5 mm/100 (Table 2).

**TABLE 2:** Pain characteristics (*N* = 295)

	Global population		DAS28‐ESR ≤ 3.2		DAS28‐ESR > 3.2		*p*‐value
	Mean or %	*SD* or *n*	Mean or %	*SD* or *n*	Mean or %	*SD* or *n*	
Current pain VAS (mm/100) (*N* = 292)	28.8	27.1	16.5	19.2	43.9	27.8	<0.0001^#^
Average last 8 days pain VAS (mm/100) (*N* = 292)	33.6	26.5	21.8	21.3	47.8	25.5	<0.0001^#^
Worse pain VAS (mm/100) (*N* = 292)	42.0	30.7	29.1	27.5	57.6	27.2	<0.0001^#^
Pain sensory qualifiers score (/100) (*N* = 275)	19.4	17.0	14.9	13.7	25.1	18.9	<0.0001^#^
Pain emotional qualifiers score (/100) (*N* = 276)	24.4	25.0	16.8	20.6	33.8	26.6	<0.0001^#^
Anxiety score (HAD) (*N* = 290)	8.4	4.5	8.1	4.5	8.7	4.5	0.2428^#^
Anxiety [11–21]	30.0%	87	26.0%	41	34.6%	46	0.2772*
Probable anxiety [8–10]	24.5%	71	25.3%	39	23.3	31	
Depression score (HAD) (*N* = 291)	5.9	3.9	5.2	3.8	6.8	3.9	0.0003^#^
Depression [11–21]	12.4%	36	7.7%	12	18.0%	24	0.0135*
Probable depression [8–10]	19.6%	57	17.4%	27	21.1%	28	
Pain interference on (0–10)							
Mood (*N* = 292)	3.2	2.7	2.5	2.5	4.1	2.6	<0.0001^#^
Walk (*N* = 291)	3.9	2.9	3.0	2.7	5.0	2.8	<0.0001^#^
Work (*n* = 293)	4.6	2.8	3.7	2.6	5.7	2.5	<0.0001^#^
Relationship (*N* = 293)	2.1	2.4	1.7	2.2	2.7	2.6	0.0008^#^
Sleep (*N* = 293)	3.3	3.2	2.3	2.6	4.7	3.2	<0.0001^#^
Enjoyment of life (*N* = 293)	2.4	2.7	2.0	2.4	2.9	2.9	0.0041^#^
Beck depression inventory (*N* = 294)	6.7	6.5	5.9	6.0	7.8	6.9	0.0180^#^
No or mild depression [0–7]	65.6%	193	70.3%	111	59.4%	79	0.0527*
Moderate to severe depression ≥ 8	34.4%	101	29.7%	47	40.6%	54	
STAI‐State (*N* = 279)	37.2	12.5	36.2	12.1	38.5	13.0	0.1305^#^
Score YA ≥ 46	26.9%	75	21.7%	33	33.1%	41	0.0342*
STAI‐Trait (*N* = 278)	40.1	11.9	39.3	11.9	41.2	11.9	0.1352^#^
Score YB ≥ 46	31.3%	87	25.5%	39	38.5%	47	0.0205*

Abbreviations: HAD, hospital anxiety depression questionnaire; STAI, state and trait anxiety inventory; VAS, visual analogue scale.

* Chi^2^ test.

^#^ Mann–Whitney test.

Among terms used by patients to describe their pain, emotional terms were more commonly cited than sensory qualifiers (24.4% ± 25.0 as against 19.4% ± 17.0 respectively). HADS scores were suggestive of clinically important anxiety or depression (54.5% with anxiety disorders; 31.9% depressive disorders), emphasized by the more accurate Beck scale (moderate or severe depression in 34.4% of cases) and STAI scores (STAIA 26.9%, STAIB 31.3%). Among our RA population, 21 patients (7.1%) had an history of depression and 16 (5,4%) were on antidepressants. In terms of the impact of pain on daily life, the most significant was the impact on working life, followed by the effects of pain on sleep and mood.

### 3.3 Subgroup analysis

#### 3.3.1 By pain score

As shown in Table 3, among patients with pain (VAS_8d_ ≥ 40 mm/100), the depression and anxiety scores, HAQ score, sensory and emotional pain qualifier scores, impact on daily life score and DAS28 score were significantly higher than in patients with a VAS < 40 mm. There was no difference between the two groups in terms of immunological status, duration of the RA or structural damage.

**TABLE 3:** Comparison of Rheumatoid arthritis patients with last 8 days average pain VAS < 40 and ≥ 40 mm/100 (*N* = 292)

	Pain VAS < 40		Pain VAS ≥ 40		*p*
	Mean or %	*SD* or *n*	Mean or %	*SD* or *n*	
Marital statut (*N* = 292)					
Single	26.7%	48	41.7%	46	0.0104*
Married or in relationship	73.3%	132	58.9%	66	
Activity (*N* = 285)					
Not working	43.7%	76	55.9%	62	0.0449*
In professional activity	56.3%	98	44.1%	49	
Disease duration (years) (*N* = 291)	13.1	9.1	13.5	10.5	0.7981^#^
ACPA or/and RF positivity (*N* = 283)	86.8	151	81.6	89	0.2420^#^
ESR (mm/h) (*N* = 289)	12.1	12.3	17.0	17.7	0.0162^#^
Number of tender joint (*N* = 292)	2.0	3.3	5.5	5.6	<0.0001^#^
Number of swollen joint (*N* = 292)	1.9	2.7	3.5	3.5	<0.0001^#^
Patient disease activity VAS (mm/100) (*N* = 292)	24.66	19.82	59.15	20.53	<0.0001^#^
DAS28‐ESR (*N* = 289)	2.5	1.1	4.0	1.3	<0.0001^#^
SENS score (*N* = 271)	21.7	20.6	21.3	18.5	0.9084^#^
HAQ score (*N* = 289)	0.9	0.6	1.5	0.7	<0.0001^#^
Steroids (*N* = 292)	36.7%	66	51.8%	58	0.011*
Pain sensory qualifiers score (/100) (*N* = 272)	12.6	11.7	30.8	18.6	<0.0001^#^
Pain emotional qualifiers score (/100) (*N* = 273)	14.2	19.0	41.8	24.5	<0.0001^#^
Anxiety score (HAD) (*N* = 287)	7.8	4.3	9.4	4.6	0.0039^#^
Depression score (HAD) (*N* = 288)	5.1	3.9	7.2	3.6	<0.0001^#^
Pain interference on (0–10)					
Mood (*N* = 289)	2.4	2.3	4.6	2.6	<0.0001^#^
Walk (*N* = 288)	2.5	2.3	6.1	2.4	<0.0001^#^
Work (*N* = 290)	3.2	2.3	6.7	2.0	<0.0001^#^
Relationship (*N* = 290)	1.6	2.0	2.9	2.7	<0.0001^#^
Sleep (*N* = 290)	2.1	2.4	5.4	3.2	<0.0001^#^
Enjoyment of life (*N* = 290)	1.8	2.2	3.4	3.0	<0.0001^#^
Beck depression inventory (*N* = 291)	5.8	5.7	8.2	7.3	0.0100^#^
STAI‐State (*N* = 276)	34.9	11.6	41.1	13.1	0.0002^#^
STAI‐Trait (*N* = 275)	38.8	11.6	42.4	12.0	0.0124^#^
Number of comorbidity (*N* = 292)	1.1	1.0	1.5	1.1	0.0029^#^

Abbreviations: ACPA, anti‐citrullinated protein antibodies (>ULN = 25 IU/ml); DAS28‐ESR, disease activity score for 28 joint counts based on the erythrocyte sedimentation rate; ESR, erythrocyte sedimentation rate; HAD, hospital anxiety depression; HAQ, Health Assessment Questionnaire from 0 to 3; RF, rheumatoid factors (>ULN = 15 IU/ml); SENS, radiographic score (simple erosion narrowing score); STAI, state and trait anxiety inventory.

* Chi^2^ test.

^#^ Mann–Whitney test.

#### 3.3.2 By DMARDs

Patients treated with sDMARDs only, those treated with bDMARDs only and those receiving a combination of both treatments did not differ in terms of quantitative and qualitative pain parameters or in relation to the impact of pain on daily life (Data S1). The results were identical with the analysis of the biological DMARDs subgroups (anti‐TNF ± sDMARDs, tocilizumab ± sDMARDs, abatacept ± sDMARDs, rituximab ± sDMARDs) (Data S1).

### 3.4 Principal component analysis (PCA) after normalization of variables

**FIGURE 2:**
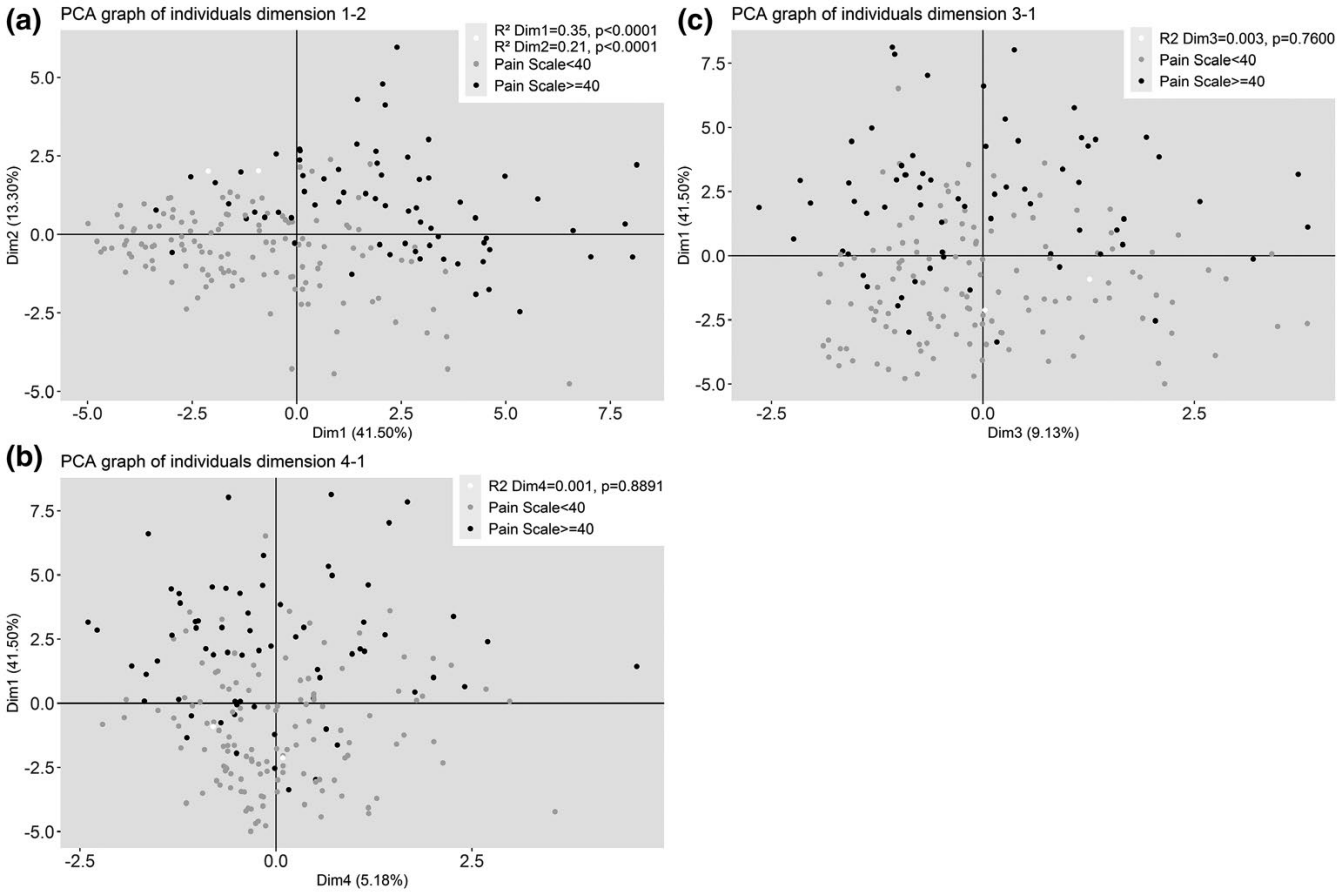
Principal component analysis – Analysis of Variance on the different patient coordinates on PCA axes in relation to their pain level (VAS < 40 mm/100 mm; VAS ≥ 40 mm/100 mm); dim 1: dimension 1 or axis 1 (emotional impact: anxiety and depression scores, sensory and emotional qualifier scores, HAQ, impact of the pain in daily life); dim 2: dimension 2 or axis 2 (Disease activity score DAS28, pain relief with current treatments); dim 3: dimension 3 (duration of RA, SENS radiographic score); dim 4: dimension 4 (ESR, DAS28) (*N* = 197)

In total, 19 variables contributed to the creation of the axes, with VAS_8d_ (<40 and ≥40 mm/100) serving as an illustrative variable. A total of 197 patients were included in the PCA analyses, these patients had non‐missing data concerning the variables included in the analysis. Four axes were retained, they represent a total variance of 70% (the 1st axis represented 41% of the variance, the 2nd 13%, the 3rd 9% and the 4th 6%) (Figure 2). The results were identical regardless of whether we took the VAS “the most intense pain in the previous 8 days” or the VAS “current pain” as illustrative variable.

The variables contributing to axis 1 were the anxiety scores (HAD, STAI A and B), the depression scores (HAD, BDI), the sensory and emotional qualifier scores, the HAQ score and the various scores for the impact of the pain on daily life. This axis, therefore, represented the emotional aspect of the pain and its impact. A high value on this axis corresponded to a high values of the corresponding variables.

The analysis of the individuals’ coordinates on axis 1 in relation to the pain level (<40, ≥40) showed that the pain level was associated with this axis (*R* ^2^ = 32%, *p* < 0.0001) As compared to mean patient coordinates, patients in the VAS_8d_ ≥ 40 group had significantly higher coordinates (*p* < 0.0001), while patients in the VAS_8d_ < 40 group had significantly lower coordinates (*p* < 0.0001) (Figure 2).

The variables contributing to axis 2 were the DAS28 and pain relief with current treatments. A high value on this axis corresponded to a high DAS28 value and a low relief intensity value.

The analysis of the individuals’ coordinates on axis 2 in relation to the pain level (<40, ≥40) showed that the pain level correlated with this axis (*R* ^2^ = 22%, *p* < 0.0001). As compared to mean patients’ coordinates, patients in the VAS_8d_ ≥ 40 group had significantly higher coordinates (*p* < 0.0001) and the patients in the VAS_8d_ < 40 group had significantly lower coordinates (*p* < 0.0001).

The variables contributing to axis 3 were the RA duration and the SENS radiographic score. The pain level was not associated with this axis (*R* ^2^ = 0,3%, *p* = 0.7749).

Axis 4 was mostly represented by the ESR and the DAS28. A high value on this axis corresponded to a high DAS28 and ESR value. The pain level was not associated with this axis (*R* ^2^ < 0.2%, *p* = 0.8023).

## 4 DISCUSSION

Among the 295 patients included, RA was generally well controlled, with more than half of patients in remission or in low disease activity. The mean VAS_8d_ was 33.6 ± 26.5 mm/100 and overall 38.4% of patients had at least moderate pain, while 13.7% had a severe pain. On the other hand, significant proportion of our population used analgesic and corticosteroid treatments. These results are better overall than those published by Taylor et al. (2010). Indeed, in this study of 879 patients undergoing biological treatment, less than 50% reported sufficient pain relief, although they judged the RA otherwise well controlled. Of 756 patients recruited in Europe, 75% reported moderate to severe pain and 60% were not satisfied with the pain intensity they were experiencing. In the 2016 literature review of Taylor et al., pain persists at an unacceptable level in patients with RA (Taylor, Moore, Vasilescu, Alvir, & Tarallo, 2016).

In our study, the patients used a higher proportion of emotional qualifiers to describe their pain compared to sensory qualifiers, highlighting the importance of the emotional factor in pain. The impact of pain on daily life affected working life most of all, followed by sleep and mood. Moreover, a high percentage of patients suffered from anxiety and/or depression. A meta‐analysis of 72 studies in 2013 and the recent study of Marrie et al. showed quite similar proportions regarding depression in RA (Marrie et al., 2018; Matcham, Rayner, Steer, & Hotopf, 2013). And multiple studies have shown a correlation between anxiety and depression and increased perception of pain (Smith & Zautra, 2008). Yet anxiety and depression evaluation are not in the core set of rheumatologic evaluation tools.

Immunological status and treatments were not different between patients with moderate to severe pain and patients with mild or no pain.

The principal component analysis led to the identification of a positive correlation between pain and anxiety/depression, but also between pain and disease activity. The functional state is also an important element, included in axis 1. The study of Häkkinen et al. have already highlighted the greater impact of pain on function, compared to radiographic damage or number of swollen and tender joints (Häkkinen et al., 2005).

A recent study of 17,006 individuals with RA reported that depressive symptoms predicted slower rates of improvement in patient‐reported pain and tender joint count, whereas depressive symptoms did not predict rates of improvement in swollen joints count and acute phase reactants (both measures of inflammation) (Rathbun, Harrold, & Reed, 2015). Depression and anxiety have an impact on the disease activity measured by the DAS28 due to their influence on the number of painful joints and the overall assessment of the patient (Matcham, Ali, Irving, Hotopf, & Chalder, 2016; Michelsen et al., 2017). In their 5‐year prospective study, Overman et al. demonstrated that psychological distress and disease activity are positively correlated (Overman et al., 2012). Thus, the association we have shown between pain and the disease activity measured by the DAS28 can be in part explained by the effect of psychological issues on the DAS28 measurement. For the axis 4, which is essentially represented by the ESR and the DAS28, there was no relation with pain, perhaps explained by the ESR weight and, thus, by the inflammation in this axis. However, the data on this axis should be treated with caution, as it only represents 6% of the total variance.

In addition, we have not shown any significant relation between pain and the duration of the disease or the articular structural damages (SENS score), which represent axis 3.

Nieuwenhuis, de Wit, Boonen, and van der Helm‐van Mil (2016) assessed the presentation of RA over the past decade. They observed less severe inflammation at presentation (lower swollen joint count, lower levels of acute phase reactants), but paradoxically, increased severity of patient reported outcomes (PROMs), especially pain, from the period 1993–1996 to the period 2011–2015. Even though inflammatory pathology is better controlled, the disease burden as experienced by patients seems to be higher.

McWilliams et al. identified subgroups of patients for which there was concordant or discordant data between patient‐reported symptoms and inflammation criteria (worse PROMs despite less markedly elevated inflammation or less severe PROMs despite elevated inflammation) (McWilliams, Ferguson, Young, Kiely, & Walsh, 2016). The authors concluded there are subgroups of patients with RA in which DAS28 might either underestimate or overestimate patients’ requirement for DMARD escalation.

There are some limitations, our study is cross‐sectional and does not allow us to assess fluctuations in pain over time. The RA patients were included in various French hospitals with a high prevalence of biologic treatments. This, therefore, limits the extent to which our results can be generalized. We have not assessed cognitive aspects of pain such as “coping” or “catastrophism”. PCA analysis was only performed with two thirds of patients because of missing data. The absence of relation between the duration of the illness, structural injuries and pain could be linked to a lack of statistical power. Others mechanisms may contribute to residual pain. Fibromyalgia has a higher prevalence in RA compared to general population. We used the ACR 1990 criteria to exclude fibromyalgia in our population, but a misclassification of putative associated fibromyalgia cannot be excluded. The second hypothesis is central sensitization. Indeed, joints affected by active synovitis are more sensitive to pain due to peripheral sensitization induced by local inflammation. Increased and sustained nociceptive input from joints, blunting of normal pain modulation and neuroinflammation with activation of microglia by cytokines and chemokines can trigger dysregulation of central nervous system named central sensitization (McWilliams & Walsh, 2017; Zhang & Lee, 2018). Activation of microglia could have active roles in the alterations in synaptic remodelling, connectivity and network function that underlie chronic pain (Ji, Nackley, Huh, Terrando, & Maixner, 2018). But, our study did not include quantitative sensory testing or pain modulation tests to explore this hypothesis.

However, we were committed to carry out a multidimensional assessment combining different quantitative and qualitative aspects, including the psychological component, and the effect on functional ability and behaviour in daily life. Moreover, the use of a descriptive statistical approach including PCA allowed us to clarify the information obtained during assessments by identifying the variables that were correlated, and by the combination of the variables into four synthetic axes.

The results of this study should have an impact on patient's daily care. More than a third of RA patients suffer from moderate to severe pain despite a high proportion of patients on biological treatment. For those patients still suffering from pain, given the strong association of pain and depression/anxiety showed in the present study, a mechanistic multidimensional analysis of the pain could avoid inappropriate therapeutic escalation of DMARDs and should offer a more tailored care. The link between anxiety/depression and persistent pain suggests a broader approach to manage these patients, in particular, include a systematic evaluation of such symptoms in the core set of rheumatologic tools. In the future, it would be interesting to develop a simplified questionnaire in order to assess various aspects of the pain in RA and to provide direction for the clinician.

## CONFLICT OF INTEREST

None.

## AUTHORS’ CONTRIBUTION

PVS and PB designed the study protocol and collected the data. PVS, SP, APT, ABL, MS, CR, RMJ, SP and PB collected the clinical data. PVS and APT wrote the manuscript. All authors discussed the results, revised the manuscript critically for important content and approved the final version.

## ETHICAL APPROVAL

French regional Ethics Committee, Comité de protection des personnes du Sud‐Ouest et Outre‐mer 4, N° CPP12‐0.007/2012‐A00058‐35.

## PATIENT CONSENT

The study complies with the Declaration of Helsinki.

## Supporting information

Data S1
