# Supplementary material for: The burden of pain in rheumatoid arthritis: Impact of disease activity and psychological factors
Source: Eur J Pain. 2020 Sep 11;24(10):1979–89. doi: 10.1002/ejp.1651 (PMC7692940; doi:10.1002/ejp.1651)
Supplement: Supplementary file 1 — Data S1 [file EJP-24-1979-s001.doc]

Data S1 : Comparison of pain characteristics by the groups of treatment

| **Characteristics of pain treatments** | | **n** | **Mean** | **SD** | **p** |
| --- | --- | --- | --- | --- | --- |
| Last 8 days average pain VAS (mm/100) | bDMARDs | 90 | 31.24 | 25.4 | 0.6076 |
|  | s & b DMARDs | 152 | 35.09 | 27.4 |  |
|  | sDMARDs | 50 | 33.6 | 26.2 |  |
| Level of patient satisfaction with pain management | bDMARDs | 89 | 76.54 | 24.1 | 0.9085 |
|  | s & b DMARDs | 146 | 78.76 | 21.7 |  |
|  | sDMARDs | 50 | 78.54 | 18.5 |  |
| Pain sensory qualifiers score (/100) | bDMARDs | 85 | 19.28 | 15.5 | 0.5553 |
|  | s & b DMARDs | 143 | 19.74 | 16.6 |  |
|  | sDMARDs | 47 | 18.79 | 20.9 |  |
| Pain emotional qualifiers score (/100) | bDMARDs | 85 | 24.87 | 25.4 | 0.9085 |
|  | s & b DMARDs | 144 | 23.13 | 23.1 |  |
|  | sDMARDs | 47 | 27.58 | 29.8 |  |
| Anxiety score (HAD) | bDMARDs | 88 | 8.83 | 4.6 | 0.5274 |
|  | s & b DMARDs | 152 | 8.14 | 4.5 |  |
|  | sDMARDs | 50 | 8.36 | 4.4 |  |
| Depression score (HAD) | bDMARDs | 89 | 6.46 | 3.7 | 0.1421 |
|  | s & b DMARDs | 152 | 5.75 | 3.9 |  |
|  | sDMARDs | 50 | 5.66 | 4.3 |  |
| Pain interference on (0-10) : - Mood | bDMARDs | 90 | 3.27 | 2.6 | 0.2072 |
|  | s & b DMARDs | 152 | 3.03 | 2.6 |  |
|  | sDMARDs | 50 | 3.86 | 3.0 |  |
| Pain interference on (0-10) : - Walk | bDMARDs | 90 | 4.07 | 2.7 | 0.5297 |
|  | s & b DMARDs | 151 | 3.91 | 3.0 |  |
|  | sDMARDs | 50 | 3.56 | 3.1 |  |
| Pain interference on (0-10) : - Work | bDMARDs | 91 | 4.99 | 2.6 | 0.2140 |
|  | s & b DMARDs | 152 | 4.39 | 2.8 |  |
|  | sDMARDs | 50 | 4.34 | 3.0 |  |
| Pain interference on (0-10) : - Relationship | bDMARDs | 91 | 2.34 | 2.3 | 0.1248 |
|  | s & b DMARDs | 152 | 1.95 | 2.5 |  |
|  | sDMARDs | 50 | 2.32 | 2.3 |  |
| Pain interference on (0-10) : - Sleep | bDMARDs | 91 | 3.37 | 3.0 | 0.9196 |
|  | s & b DMARDs | 152 | 3.37 | 3.2 |  |
|  | sDMARDs | 50 | 3.24 | 3.3 |  |
| Pain interference on (0-10) :- Enjoyment of life | bDMARDs | 91 | 2.73 | 2.7 | 0.2324 |
|  | s & b DMARDs | 152 | 2.2 | 2.7 |  |
|  | sDMARDs | 50 | 2.48 | 2.7 |  |
| Health Assessment Questionnaire-Disability Index (HAQ-DI) | bDMARDs | 91 | 1.24 | 0.7 | **0.0023** |
|  | s & b DMARDs | 151 | 1.14 | 0.7 |  |
|  | sDMARDs | 50 | 0.82 | 0.7 |  |
| Beck depression inventory | bDMARDs | 91 | 7.1 | 5.7 | 0.2283 |
|  | s & b DMARDs | 154 | 6.52 | 6.7 |  |
|  | sDMARDs | 49 | 6.65 | 7.3 |  |
| Intensity of relief of pain | bDMARDs | 81 | 72.52 | 22.6 | 0.8548 |
|  | s & b DMARDs | 125 | 71.94 | 24.3 |  |
|  | sDMARDs | 46 | 73.07 | 24.9 |  |
| **With détails of Biologic DMARDs** |  |  |  |  |  |
| Last 8 days average pain VAS (mm/100) | sDMARDs only | 50 | 33.6 | 26.2 | 0.2645 |
|  | AntiTNF | 53 | 34.42 | 24.3 |  |
|  | Tocilizumab | 91 | 30.2 | 27.1 |  |
|  | Abatacept | 58 | 33.21 | 26.7 |  |
|  | Rituximab | 40 | 41.18 | 28.1 |  |
| Level of patient satisfaction with pain management | sDMARDs only | 50 | 78.54 | 18.5 | 0.8516 |
|  | AntiTNF | 53 | 79.92 | 19.5 |  |
|  | Tocilizumab | 88 | 76.78 | 23.7 |  |
|  | Abatacept | 55 | 79.6 | 22.2 |  |
|  | Rituximab | 39 | 75.38 | 24.6 |  |
| Pain sensory qualifiers score (/100) | sDMARDs only | 47 | 18.79 | 20.9 | 0.7378 |
|  | AntiTNF | 51 | 20.64 | 16.6 |  |
|  | Tocilizumab | 88 | 20.33 | 17.2 |  |
|  | Abatacept | 55 | 17.17 | 13.3 |  |
|  | Rituximab | 34 | 19.85 | 17.5 |  |
| Pain emotional qualifiers score (/100) | sDMARDs only | 47 | 27.58 | 29.8 | 0.2743 |
|  | AntiTNF | 51 | 30.22 | 25.5 |  |
|  | Tocilizumab | 89 | 22.67 | 23.3 |  |
|  | Abatacept | 55 | 20.26 | 23.1 |  |
|  | Rituximab | 34 | 22.69 | 23.8 |  |
| Anxiety score (HAD) | sDMARDs only | 50 | 8.36 | 4.4 | 0.8579 |
|  | AntiTNF | 53 | 8.34 | 4.2 |  |
|  | Tocilizumab | 91 | 8.74 | 4.5 |  |
|  | Abatacept | 56 | 8.16 | 4.5 |  |
|  | Rituximab | 40 | 8 | 5.1 |  |
| Depression score (HAD) | sDMARDs only | 50 | 5.66 | 4.3 | 0.8581 |
|  | AntiTNF | 53 | 6.42 | 4.3 |  |
|  | Tocilizumab | 92 | 6.02 | 3.7 |  |
|  | Abatacept | 56 | 5.82 | 3.7 |  |
|  | Rituximab | 40 | 5.73 | 3.9 |  |
| Pain interference on (0-10) : - Mood | sDMARDs only | 50 | 3.86 | 3.0 | 0.2779 |
|  | AntiTNF | 54 | 3.35 | 2.5 |  |
|  | Tocilizumab | 90 | 2.96 | 2.5 |  |
|  | Abatacept | 58 | 2.84 | 2.6 |  |
|  | Rituximab | 40 | 3.55 | 2.9 |  |
| Pain interference on (0-10) : - Walk | sDMARDs only | 50 | 3.56 | 3.1 | 0.6272 |
|  | AntiTNF | 54 | 3.94 | 3.0 |  |
|  | Tocilizumab | 90 | 3.8 | 2.8 |  |
|  | Abatacept | 58 | 3.88 | 2.8 |  |
|  | Rituximab | 39 | 4.51 | 2.9 |  |
| Pain interference on (0-10) : - Work | sDMARDs only | 50 | 4.34 | 3.0 | 0.9481 |
|  | AntiTNF | 54 | 4.72 | 2.5 |  |
|  | Tocilizumab | 91 | 4.58 | 2.7 |  |
|  | Abatacept | 58 | 4.5 | 3.0 |  |
|  | Rituximab | 40 | 4.7 | 2.8 |  |
| Pain interference on (0-10) : - Relationship | sDMARDs only | 50 | 2.32 | 2.3 | 0.8441 |
|  | AntiTNF | 54 | 2.07 | 2.4 |  |
|  | Tocilizumab | 91 | 2.15 | 2.4 |  |
|  | Abatacept | 58 | 2.12 | 2.6 |  |
|  | Rituximab | 40 | 1.98 | 2.5 |  |
| Pain interference on (0-10) : - Sleep | sDMARDs only | 50 | 3.24 | 3.3 | 0.6024 |
|  | AntiTNF | 54 | 3.87 | 3.2 |  |
|  | Tocilizumab | 91 | 3.03 | 3.0 |  |
|  | Abatacept | 58 | 3.24 | 3.2 |  |
|  | Rituximab | 40 | 3.65 | 3.1 |  |
| Pain interference on (0-10) : - Enjoyment of life | sDMARDs only | 50 | 2.48 | 2.7 | 0.9285 |
|  | AntiTNF | 54 | 2.65 | 3.0 |  |
|  | Tocilizumab | 91 | 2.08 | 2.3 |  |
|  | Abatacept | 58 | 2.31 | 2.6 |  |
|  | Rituximab | 40 | 2.93 | 3.3 |  |
| Health Assessment Questionnaire-Disability Index (HAQ-DI) | sDMARDs only | 50 | 0.82 | 0.7 | **0.0195** |
|  | AntiTNF | 53 | 1.11 | 0.7 |  |
|  | Tocilizumab | 92 | 1.21 | 0.7 |  |
|  | Abatacept | 57 | 1.2 | 0.7 |  |
|  | Rituximab | 40 | 1.18 | 0.7 |  |
| Beck depression inventory | sDMARDs only | 54 | 8.3 | 8.3 | 0.7141 |
|  | AntiTNF | 49 | 6.65 | 7.3 |  |
|  | Tocilizumab | 93 | 6.3 | 5.3 |  |
|  | Abatacept | 58 | 6.41 | 6.1 |  |
|  | Rituximab | 40 | 6.1 | 5.5 |  |
| Intensity of relief of pain | sDMARDs only | 46 | 73.07 | 24.9 | 0.8558 |
|  | AntiTNF | 47 | 71.32 | 23.6 |  |
|  | Tocilizumab | 82 | 72.67 | 22.9 |  |
|  | Abatacept | 45 | 73 | 26.0 |  |
|  | Rituximab | 32 | 70.97 | 23.1 |  |

DMARDS : disease modifying anti-rheumatic drugs ; sDMARDs : synthetic DMARDS ; bDMARDS : biologic DMARDS ;

s & b DMARDS : synthetic and biologic DMARDS ; HAQ: Health Assessment Questionnaire from 0 to 3; VAS: visual analogue scale; HAD: hospital anxiety depression questionnaire.
